# Plasma free hemoglobin is associated with LDH, AST, total bilirubin, reticulocyte count, and the hemolysis score in patients with sickle cell anemia

**DOI:** 10.1007/s00277-025-06253-w

**Published:** 2025-02-19

**Authors:** Angela Liu, Charleen Jacobs-McFarlane, Paola Sebastiani, Jeffrey Glassberg, Sarah McCuskee, Susanna Curtis

**Affiliations:** 1 Department of Hematology and Medical Oncology, Icahn School of Medicine at Mount Sinai, New York, NY USA; 2 Department of Emergency Medicine, Icahn School of Medicine at Mount Sinai, New York, NY USA; 3 Institute for Clinical Research and Health Policy Studies, Tufts Medical Center, Boston, MA USA

**Keywords:** Sickle cell disease, Hemolysis, Plasma free hemoglobin, Hemolysis score

## Abstract

Plasma free hemoglobin (PFH) is a direct biomarker for hemolysis that has been associated with clinical complications such as pulmonary hypertension and death in patients with sickle cell disease (SCD). We sought to characterize the relationship between PFH and more clinically available hemolytic markers including lactate dehydrogenase (LDH), aspartate aminotransferase (AST), bilirubin, reticulocyte percentage and to derive a composite hemolysis score derived from principal component analysis (PCA) of these biomarkers. In 68 adult patients (median age 31 years old, IQR 25–39) with HbSS or HbSβ^0^-thalassemia enrolled in the IMPROVE II study, median PFH was elevated at 21.9 mg/dL (IQR 9.9–44.9 mg/dL) at steady state. Using Pearson correlation analysis, PFH had a stronger relationship to LDH (*R* = 0.699), AST (*R* = 0.587), and total bilirubin (*R* = 0.475), compared to reticulocyte count (*R* = 0.316). The hemolysis score was significantly associated with PFH (*R* = 0.677). When compared with other laboratory measures, PFH correlated with hemoglobin (*R*= -0.275) and HbS (*R* = 0.277), but did not correlate with white blood cell count (WBC) or HbF. The hemolysis score was significantly associated with WBC (*R* = 0.307), hemoglobin (*R* = -0.393), HbF (*R*=- 0.424), and HbS (*R* = 0.423). This study confirms that the conventional hemolytic biomarkers LDH, AST, bilirubin, and reticulocyte percentage correlate with PFH. Additionally, the hemolysis score is a valid tool to measure hemolysis and that it may be a marker of global hemolysis as opposed to PFH, which quantifies intravascular hemolysis. Further studies will be needed to elucidate the role of PFH and intravascular hemolysis in the development of clinical complications of sickle cell disease. Statements and Funding Declarations: The research leading to these results received funding from the National Heart, Lung, and Blood Institute (NHLBI) R01 HL142671 Grant under J.G. J.G. has also served as a consultant for CSL Behring, Novartis, and Novo Nordisk synteract DSMB and is supported by NHLBI RO1HL159116, R01 HL142671, R01 ES030717, UG1 HL138645, UH3 HL143192, U01HL167036, and the Doris Duke Charitable Foundation Advancing Cures grant. S.C. has served as a consultant for Pfizer and is supported by the NHLBI 5K23HL151884 grant. A.L. is supported by the NHLBI 5T32HL129974-05. C.J.M is supported by the NHLBI 5T32HL129974-05. P.S., and S.M. declare no conflicts and/or funding.

## Introduction

Sickle cell disease (SCD) is an inherited blood disorder characterized by vaso-occlusion, hemolysis, and chronic inflammation [1]. In patients with SCD, the degree of hemolysis is associated with complications of various organs including the lungs, brain, and kidneys [2, 3]. Markers of hemolysis have been shown to increase during pain crises [4] and be improved in response to sickle cell therapies such as chronic transfusions [5]. Furthermore, sickle cell patients with more chronic hemolysis have been found to be at increased risk for devastating sequelae including pulmonary hypertension, priapism, leg ulcers, vaso-occlusive crises (VOC), and death [6–8]. Thus, it is important to characterize the degree of hemolysis to better risk stratify sickle cell patients and monitor for complications and response to therapy over time.

Plasma free hemoglobin (PFH) is one of the most specific biomarkers of intravascular hemolysis [9]. Free hemoglobin is released when a red blood cell hemolyzes. Free heme can cause oxidative damage, contributing to the development of the complications above [6]. The downside of this test is that its turnaround time is on the order of days, limiting its clinical utility for prompt decision making. Lactate dehydrogenase (LDH), aspartate aminotransferase (AST), bilirubin, and reticulocyte percentage are other biomarkers that are indirect surrogates of hemolysis but are more readily available and often used to estimate hemolysis in patients with SCD [6]. The relationship between PFH and other hemolytic markers in SCD has not been well established. Kato et al. [10] demonstrated that PFH is associated with LDH in SCD but there have been no other studies evaluating the relationship between PFH and AST, bilirubin, and reticulocyte percentage. It is important to confirm that these biomarkers are all correlated to PFH and the strength of these associations.

A composite index called the hemolysis score, which is derived from principal component analysis of LDH, AST, total bilirubin, and reticulocyte percentage has been previously validated as a surrogate to estimate the degree of hemolysis in patients with SCD [11]. Because LDH, AST, bilirubin, and reticulocyte percentage are indirect markers of hemolysis that can be affected by other factors, the hemolysis score is a useful index that incorporates all four hemolytic biomarkers and accounts for as much of the variance as possible. There has been only one study to our knowledge to evaluate the relationship between PFH and the hemolysis score. Nouraie et al. showed that PFH was significantly higher in the highest hemolysis score quartile when compared to that of the lowest quartile [2]. Although this paper established the differences in the highest and lowest quartiles of the hemolysis score, it did not describe the distribution and relationship to PFH in the other quartiles.

Given the lack of data in this area, we sought to investigate PFH and its relationship with clinically available biomarkers (LDH, AST, total bilirubin, indirect bilirubin, reticulocyte percentage, and absolute reticulocyte count) and the hemolysis score derived from a combination of these biomarkers in patients with sickle cell.

## Methods

Data from the IMPROVE II study [12] were used. IMPROVE II was a randomized, placebo-controlled, single-center interventional trial at our institution. The study was conducted in accordance with the Declaration of Helsinki and the protocol was approved by the Mount Sinai Health System institutional review board (IRB). Informed consent was obtained from all patients. Patients with SCD without asthma were randomized to receive either mometasone furoate 220 mcg or placebo inhaled powder for 48 weeks. Patients were adults (age ≥ 18 years old) confirmed to have either HbSS or HbSβ⁰-thalassemia. 

Baseline laboratory testing at the first visit prior to intervention was used in the current study. The patients were in steady state and not experiencing a vaso-occlusive crisis (VOC). Pearson correlation analysis was used to evaluate the relationship between PFH and the variables AST, LDH, reticulocyte percent (retic), and total bilirubin (Tbili). Absolute reticulocyte count (ARC) and indirect bilirubin were also analyzed.

The hemolysis score was derived from the first component of the principal component analysis (PCA) of the log-standardized values of AST, LDH, retic, and Tbili using the SPSS software package [13]. PCA of all four variables, as well as combinations of two or three variables, were derived. These hemolysis scores were then compared to PFH using Pearson correlation analysis.

Pearson correlation coefficients were calculated to compare PFH and hemolysis to other laboratory data associated with disease severity including white blood cell (WBC) count, hemoglobin (Hgb), hemoglobin F (HbF) and hemoglobin S (HbS) percentages.

PFH and the hemolysis score were stratified by hydroxyurea use. White blood cell (WBC) count, hemoglobin (Hgb), hemoglobin F (HbF) were also stratified by hydroxyurea use to establish baseline hematologic characteristics. Medians were compared using the Mann-Whitney U test.

## Results

There were 68 sickle cell patients with complete, steady-state laboratory data, 89.7% of whom were HbSS and 10.3% of whom were HbSβ⁰-thalassemia. Demographics of the patients are in Table 1. The median age was 31 years old (IQR 25–39). The median PFH was 21.9 mg/dL (IQR 9.9–44.9 mg/dL). Median values of each variable AST, LDH, total bilirubin, indirect bilirubin, reticulocyte percentage, and absolute reticulocyte count were higher than the normal range (Table 1).

**Table 1:** Baseline demographics and laboratory findings of included individuals

Baseline demographics		Value	
N		68	
Age (y), median (IQR)		31 (25–39)	
Male sex, *n* (%)		36 (52.9%)	
HbSS genotype, *n* (%)		61 (89.7%)	
Laboratory findings	**N**	**Value**	**Normal values**
Plasma free hemoglobin (mg/dL)	68	21.9 (9.9–44.9)	0.0–4.9
Aspartate aminotransferase (U/L)	68	35 (29–48)	1–35
Lactate Dehydrogenase (U/L)	68	416 (311–574)	100–220
Total bilirubin (mg/dL)	68	3.3 (1.9–4.1)	0.1–1.2
Bilirubin, indirect (mg/dL)	65	2.4 (1.3–3.5)	0.2–0.8
Reticulocyte (%)	66	7.4 (4.5–9.7)	0.7–2.8
Absolute reticulocyte count (cells/uL)	66	188.5 (123.4–248.9)	50–100
White blood cell count (x10^3^/uL)	68	8.9 (7.1–11.2)	4.5–11.0
Hemoglobin (g/dL)	68	8.8 (7.8–9.6)	13.9–16.3
HbS (%)	67	80 (74–85)	
HbF (%)	67	13 (5–17)	

Laboratory findings are expressed as median (IQR)

To evaluate the relationship between PFH and other markers of hemolysis, each variable was compared to PFH (Table 2). PFH was significantly associated with LDH (*R* = 0.699, *p* < 0.001), AST (*R* = 0.587, *p* < 0.001), total bilirubin (*R* = 0.475, *p* < 0.001), and reticulocyte count (*R* = 0.316, *p* = 0.010). Indirect bilirubin was evaluated and was also associated with PFH (*R* = 0.454, *p* < 0.001). ARC did not correlate with PFH (*R* = 0.241, *p* = 0.051).

**Table 2:** Relationship between plasma free hemoglobin and indirect markers of hemolysis

	Correlation coefficient	*p*-value
Individual variable		
LDH	0.699	< 0.001
AST	0.587	< 0.001
Total bilirubin	0.475	< 0.001
Bilirubin, indirect	0.454	< 0.001
Reticulocyte	0.316	0.010
Absolute reticulocyte count	0.241	0.051
Hemolysis score (variables used in PCA)		
AST, LDH, Tbili, Retic	0.677	< 0.001
AST, LDH, Tbili	0.706	< 0.001
AST, LDH, Retic	0.704	< 0.001
LDH, Tbili, Retic	0.610	< 0.001
AST, Tbili, Retic	0.507	< 0.001
AST, LDH	0.709	< 0.001
LDH, Tbili	0.658	< 0.001
LDH, Retic	0.639	< 0.001
AST, Tbili	0.632	< 0.001
AST, Retic	0.557	< 0.001
Tbili, Retic	0.444	< 0.001

Abbreviations: *AST* aspartate aminotransferase, *LDH* lactate dehydrogenase, *Retic* reticulocyte count, *Tbili* total bilirubin, *ARC* absolute reticulocyte count

The hemolysis score was derived from the first component of PCA of the four variables AST, LDH, Tbili, and reticulocyte. This first component explained 60.0% of the total variance among the four variables in the sample. This hemolysis score was significantly associated with PFH with an R-value of 0.677, *p* < 0.001. PCA of various combinations of two or three variables were evaluated (Table 2). The hemolysis score that most strongly correlated with PFH was derived from two variables - AST and LDH (*R* = 0.709, *p* < 0.001).

**Fig. 1:**
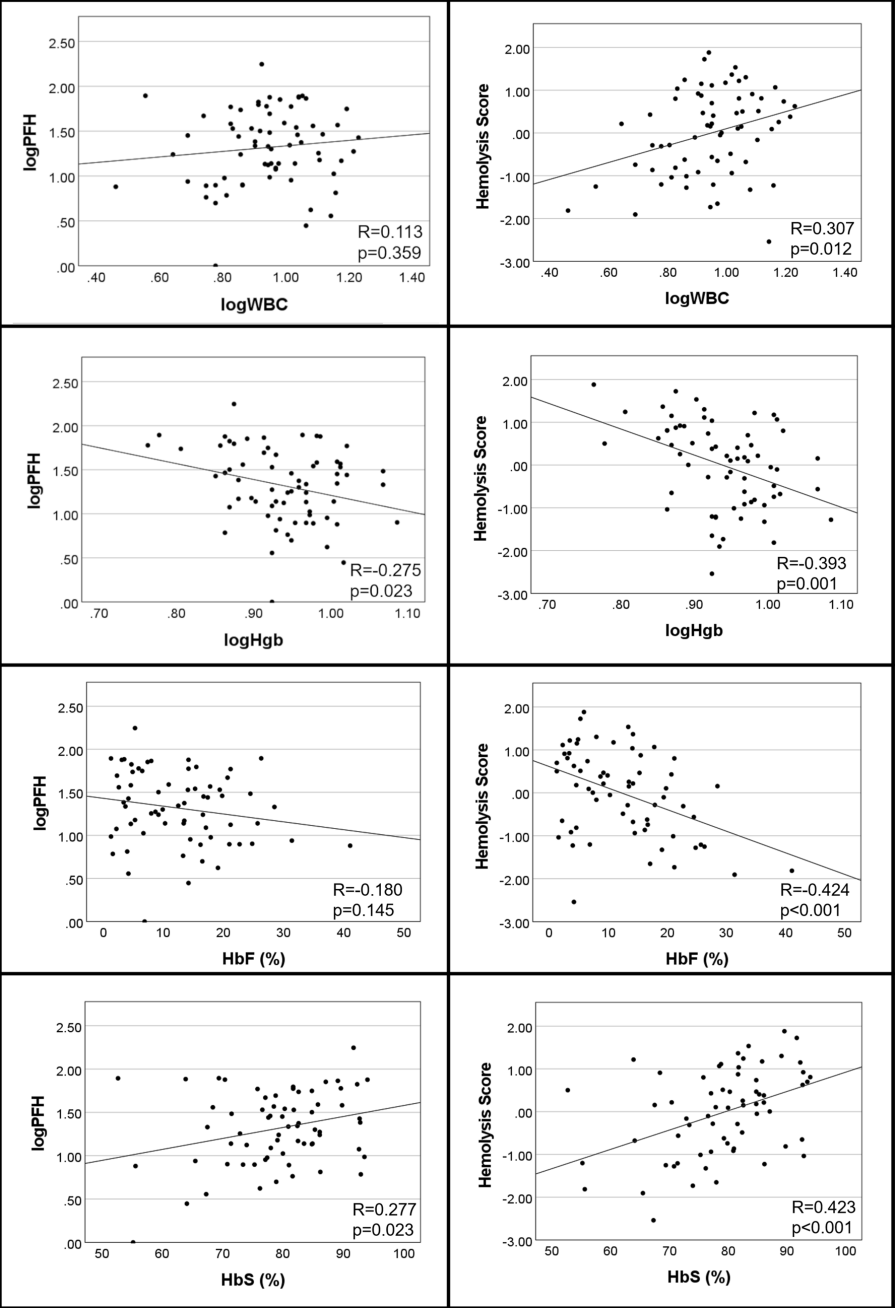
Relationship of plasma free hemoglobin (PFH) and the 4-variable hemolysis score (AST, LDH, Tbili, Retic) with other laboratory features. Abbreviations: AST, aspartate aminotransferase; Bilirubin, total bilirubin; HbF, fetal hemoglobin; HbS, sickle hemoglobin; Hgb, hemoglobin; LDH, lactate dehydrogenase; PFH, plasma free hemoglobin; Retic, reticulocyte count; WBC, white blood cell count

The relationship between PFH or the hemolysis score and other laboratory features was examined (Fig. 1). PFH correlated with hemoglobin (*R*= −0.275, *p* = 0.023) and HbS (*R* = 0.277, *p* = 0.023), but did not correlate with WBC or HbF. The hemolysis score was associated with WBC (*R* = 0.307, *p* = 0.012), hemoglobin (*R* = −0.393, *p* = 0.001), HbF (*R*= −0.424, *p* < 0.001), and HbS (*R* = 0.423, *p* < 0.001).

**Fig. 2:**
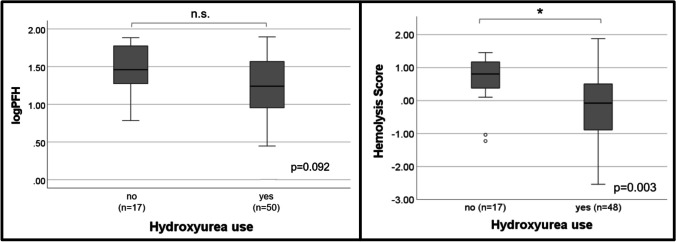
PFH and the 4-variable hemolysis score (AST, LDH, Tbili, Retic) stratified by hydroxyurea use. Note: Hydroxyurea use was missing in 1 patient. Hemolysis score was unable to be calculated in 2 patients

PFH and the hemolysis score were stratified by hydroxyurea status (Fig. 2). Hemoglobin, hemoglobin F, and white blood cell count were not significantly different between those treated with hydroxyurea and those not treated with hydroxyurea (Table 3). Patients taking hydroxyurea had lower PFH compared to patients not taking hydroxyurea (17.4 mg/dL vs. 28.8 mg/dL, respectively), however, this did not reach statistical significance (*p* = 0.092). The hemolysis score was lower in the hydroxyurea group compared to the non-hydroxyurea group (−0.08 vs. 0.81, respectively), however, this difference was statistically significant (*p* = 0.003).

**Table 3:** Hematologic values stratified by hydroxyurea use

	Untreated	Hydroxyurea treated	*p*-value
*N*	17	50	
Hemoglobin (g/dL)	8.4 (7.4–9.3)	9.0 (8.2–10.0)	0.624
HbF (%)	8.0 (3.5–13.8)	14.2 (6.6–20.1)	0.091
White blood cell count (x10^3^/uL)	10.9 (8.6–12.2)	8.8 (6.6–10.5)	0.057

Lab values listed as median (IQR)

## Discussion

We sought to evaluate the relationship between PFH and other, more easily and quickly obtainable markers of hemolysis. LDH is associated with PFH in patients with sickle cell disease [10], however, the relationship between PFH and the other biomarkers has not been established.

Our study demonstrated that AST, LDH, total bilirubin, indirect bilirubin, and reticulocyte count correlated with PFH in patients with SCD. PFH is thought to be a direct biomarker of intravascular hemolysis as it is released from the cell during the process [9]. During intravascular hemolysis, a red blood cell releases hemoglobin, LDH, and AST [6]. Our findings support this model of intravascular hemolysis, as the PCA of these two laboratory parameters (LDH and AST) most closely correlated to PFH. It is important to note that there are limitations as AST, LDH, and bilirubin are indirect markers of hemolysis and can be affected by other events and conditions outside of hemolysis. LDH can be elevated in other conditions with tissue injury [14] and AST can be affected by muscle and liver injury [15]. Thus, both LDH and AST are not specific to hemolysis. Bilirubin is also a nonspecific surrogate marker for hemolysis as it is the converted form of free heme and can also be affected by liver or biliary disease [6, 16]. Elevated PFH has been linked with sickle cell complications including pulmonary hypertension, cerebral vasculopathy, and increased risk of death [5, 17, 18], making it a clinically important biomarker to study. PFH is also easier to measure than other direct markers of hemolysis such as RBC survival [19]. To our knowledge, this is the first study quantifying the direct relationship between PFH and AST, bilirubin, and reticulocyte count in patients with SCD. Moreover, given that a PCA of LDH and AST most strongly correlated with PFH, perhaps LDH and AST could further evaluated as potential biomarkers of intravascular hemolysis. This would be clinically useful as more SCD drugs targeting hemolysis are being developed.

Interestingly, reticulocyte count had a weaker correlation with PFH compared to the other hemolytic markers (AST, LDH, bilirubin). In SCD, both extravascular and intravascular hemolysis occur, and it is thought that only one-third of hemolysis in patients with SCD is intravascular [20]. Reticulocyte count is considered one of the most robust markers of hemolysis as it is reflective of red cell production, which increases during intravascular as well as extravascular hemolysis [16]. PFH, which is a marker of only intravascular hemolysis, may not reflect the total extent of hemolysis [6]. This likely explains why PFH does not correlate as strongly to reticulocyte count as it does not reflect extravascular hemolysis.

The hemolysis score derived in our study incorporated LDH, AST, bilirubin, and reticulocyte levels and thus may capture both intravascular as well as extravascular hemolysis. In research and clinical care, it can be useful to consolidate multiple, collinear markers of hemolysis into a single score. We found that the hemolysis score correlated with PFH. Our 4-variable hemolytic score PCA yielded a primary component which explained a similar proportion of the total variance to the hemolytic components in the literature (64.1% in our study vs. 51–67% in other studies) [2, 11, 13, 21–24]. The study done by Nouraie et al. is the only paper to our knowledge to evaluate the relationship between PFH and hemolysis score (derived from AST, LDH, Tbili, reticulocyte count), which support our findings [2]. The authors showed that cell-free hemoglobin was higher in the highest quartile of their derived hemolytic score when compared to the lowest quartile. However, it was unclear how the hemolysis score compared to PFH in the other quartiles. Our study used all hemolysis score values and found that they correlated to PFH, which was not known previously.

In evaluating the relationship between hemolysis and other laboratory features, we found that PFH was only associated with lower hemoglobin and higher HbS. Hydroxyurea use was associated with a lower PFH numerically but was not statistically significant. These findings may be limited by the small number of patients in our study, as a previous study showed that HbSS individuals on hydroxyurea therapy had significantly lower LDH and plasma free hemoglobin [10].

We found that the hemolysis score was associated with higher HbS percentage, and lower hemoglobin and HbF percentage. Hydroxyurea use was associated with a lower hemolysis score. These findings were also found in the Nouriae study [2]. This is the first study to assess the relationship between the hemolysis score and WBC count and we found a significant association. This may be the case as both a higher hemolysis score and leukocytosis are associated with sickle cell severity [25].

Our findings may suggest that the hemolysis score which incorporates four hemolytic biomarkers that span intravascular and extravascular hemolysis, in contrast to PFH which reflects only intravascular hemolysis, may be a more robust marker of overall ‘blood injury’ and disease severity. The hemolysis score can also be determined as soon as LDH, AST, Tbili, and reticulocyte become available, potentially making this a more clinically useful measure of hemolysis compared to PFH, which may take days to return. Future studies are indicated to investigate the predictive value of the hemolysis score and its validity as a marker of disease severity and response to therapy. One limitation of our study is a small sample size, though this is due to the prospective nature of the data. Given that laboratory and clinical data were collected longitudinally over the course of 48 weeks in the IMPROVE II trial, future studies will be aimed at investigating whether the hemolysis score and PFH can be trended over time, and the impact of those changes on clinical outcomes such as VOC and organ injury. Another potential application of PFH and the hemolysis score would be to evaluate its validity during an acute VOC and its predictive value on clinical outcomes. Generalizability is another limitation of this study. The hemolysis score requires statistical support to compute, which may not be accessible at all health facilities. Thus, future studies should be aimed at establishing a clinically relevant scoring system using these readily available hemolytic labs without the need of advanced statistical techniques.

## Conclusion

Our study showed that PFH measured at baseline in patients with sickle cell disease was associated with other hemolytic markers LDH, AST, total bilirubin, and reticulocyte count. Intuitively, PFH was also associated with the hemolysis score which was derived from the first component of a principal component analysis of these hemolytic markers. PFH had a stronger relationship to LDH, AST, and bilirubin compared to reticulocyte percentage, suggesting that PFH may be more specific to intravascular hemolysis. These findings elucidate the relationship between PFH and other hemolytic biomarkers. We would recommend using AST and LDH to estimate the degree of intravascular hemolysis, and using the 4-component hemolysis score which incorporates AST, LDH, reticulocyte count, and total bilirubin to evaluate the severity of global hemolysis. Further studies will be needed to evaluate these relationships over time in both the acute and chronic settings, and in relation to clinical sequelae of sickle cell disease.

## Data Availability

No datasets were generated or analysed during the current study.
